# Scoping review of public health emergency management training programmes and curricula in Africa (2000–2025): implications for policy, practice and research

**DOI:** 10.1136/bmjph-2026-004980

**Published:** 2026-09-21

**Authors:** Olushayo Oluseun Olu, Peter Adewuyi, Henry Kyobe Bosa, Julius Monday, Saah Tamba Alpha, Rhoda Elizabeth King, Kwuakuan Dorh Monnea Yealue, Elton Tichapiwa Tanyanyiwa, Oluyemisi Akinwande, Sheick O Coulibaly, Mohamed Ismail, Sylvester Maleghemi, Amos Petu, Abdulmumini Usman, Robert Lubajo

**Affiliations:** 1 World Health Organization Regional Office for Africa, Brazzaville, Congo; 2 African Field Epidemiology Network Programme, Monrovia, Montserrado, Liberia; 3 Outbreaks and Epidemics, Interdisciplinary Consortium for Epidemics Research, Kampala, Uganda; 4 Incident Management Team, Ministry of Health, Kampala, Uganda; 5 Emergency Preparedness and Response Programme, World Health Organization Country Office, Monrovia, Montserrado, Liberia; 6 Programme Management Office, World Health Organization Country Office, Monrovia, Montserrado, Liberia; 7 Independent Global Health Consultant, Washington, District of Columbia, USA; 8 Laboratory and Diagnostics Programme, World Health Organization Regional Office for Africa, Brazzaville, Congo; 9 Essential Medicines Programme, World Health Organization Regional Office for Africa, Brazzaville, Congo; 10 Vaccine Preventable Diseases Programme, World Health Organization Headquarters, Geneva, Switzerland; 11 Global Health Planning, Program, Economics and Finance Group, Ilofa, Kwara, Nigeria; 12 Public Health Disaster Risk Management Consultant, Kano, Nigeria

**Keywords:** Emergencies, Education, Medical, Public Health

## Abstract

**Objectives:**

This study mapped the landscape of public health emergency management (PHEM) training programmes and curricula in Africa from 2000 to 2025 to identify trends, strengths and persistent gaps.

**Design:**

The study was conducted following the Arksey and O’Malley framework, refined by Levac *et al*, and reported in line with the Preferred Reporting Items for Systematic Reviews and Meta-Analyses Extension for Scoping Reviews (PRISMA-ScR) guidelines.

**Data sources:**

A comprehensive search of peer-reviewed databases, including Ovid MEDLINE, PubMed, Scopus and CINAHL, was conducted in September 2025. Additional non-indexed resources were obtained from organisational repositories and websites of regional public health organisations, *African Journals Online* and Google Scholar.

**Eligibility criteria:**

Peer-reviewed articles, technical reports, theses and organisational publications published between 2000 and 2025 that described the design, delivery, implementation or evaluation of PHEM training programmes in Africa were included in the study.

**Data extraction and synthesis:**

Data were extracted using a standardised form capturing programme characteristics, competencies, delivery modalities, evaluation methods, outcomes and challenges. Qualitative data were analysed thematically and mapped against the review objectives.

**Results:**

Of 334 records identified, 32 met the inclusion criteria. Training initiatives ranged from short courses to fellowships and degree programmes, with a predominant focus on epidemic management. Critical domains such as leadership, humanitarian response, One Health, diagnostics and gender equity were under-represented. Few programmes reported formal competency assessments or long-term evaluation outcomes. Key challenges identified included reliance on donor funding, weak institutional ownership, poor integration with academic systems and limited sustainability. Emerging needs included training in the Prevention of Sexual Exploitation, Abuse and Harassment, the use of digital health technologies and integration of the humanitarian-development-peace nexus.

**Conclusion:**

PHEM training in Africa remains heavily oriented towards epidemic response, with insufficient emphasis on complex emergencies. Strengthening workforce resilience requires comprehensive and sustainably financed curricula.

**WHAT IS ALREADY KNOWN ON THIS TOPIC**

- Africa continues to face recurrent infectious disease outbreaks and complex humanitarian crises that overwhelm its fragile health systems.
- A competent public health workforce is critical for effective public health emergency management, yet major capacity gaps persist across the continent.
- Several regional training initiatives, such as the Field Epidemiology Training Programs and the African Volunteer Health Corps-Strengthening and Utilizing Response Groups for Emergencies, have been implemented, but their scope and integration vary widely across countries.

**WHAT THIS STUDY ADDS**

- This study provides the first comprehensive mapping of public health emergency management training initiatives and curricula across all 54 African countries between 2000 and 2025.
- It reveals that most programmes focus on epidemic response, with limited inclusion of competencies for public health management in humanitarian emergencies or emerging issues such as the application of innovative digital health technologies, gender equity, prevention of sexual exploitation and abuse and the concept of humanitarian-development-peace nexus.
- It identifies critical gaps in evaluation methods, sustainability and institutional integration that undermine long-term workforce capacity building efforts.

**HOW THIS STUDY MIGHT AFFECT RESEARCH, PRACTICE OR POLICY**

- The study identified several evidence gaps that warrant further investigation. These include, among others, the need to define public health emergency management competencies considering the evolving landscape of public health emergencies and humanitarian crises and exploration of the potential applications of artificial intelligence in public health emergency management training programmes in Africa.

## Introduction

 Africa faces a rising frequency and diversity of public health emergencies that strain its fragile health systems and increase disease burden. Between 2001 and 2022, over 2200 health events were recorded,^1^ the majority of which were infectious disease outbreaks, including Ebola virus disease (EVD)^2^ and cholera,^3^ which have caused tens of thousands of deaths. These challenges are worsened by humanitarian crises and climate-related threats such as floods and droughts, with significant economic losses.^4,5^ Emergencies disrupt essential services and development gains, while severely affecting healthcare workers through infection risks, burnout and psychological stress.^6^ Damage to facilities and supply chains further undermines effective health service delivery across the continent.^7^ Thus, the importance of timely and effective public health emergency management (PHEM) in safeguarding population health towards the attainment of the Sustainable Development Goals and Sendai Framework for Disaster Risk Reduction in Africa cannot be overemphasised.^8^

Timely and effective PHEM depends on resilient health systems with the capacity to prepare for, respond to and recover from health crises.^9^ A skilled and competent health workforce is central to this resilience, enabling the anticipation, detection and control of public health emergencies while preventing nosocomial infections and deaths.^6^ However, such capacity remains limited in Africa. For instance, the 2014–2016 West African Ebola outbreak exposed critical workforce gaps that delayed response efforts.^10^ Conversely, structured PHEM training programmes have been demonstrated to improve outbreak surveillance and response capacities, underscoring their importance for sustainable development and health security in Africa.^11^

Over the past two decades, global and regional initiatives have been developed and implemented to strengthen PHEM workforce capacity. The WHO Regional Office for Africa (WHO/AFRO) developed and piloted PHEM competencies in 2018.^12^ In 2023, WHO/AFRO and Africa Centres for Disease Control and Prevention (Africa CDC) launched the African Volunteer Health Corps- Strengthening and Utilizing Response Groups for Emergencies initiative to enable rapid emergency response within 24–48 hours^13,14^. Africa CDC’s New Public Health Order also prioritised workforce development through fellowships, training centres and competency-based curricula.^15,16^ Additionally, US-supported Field Epidemiology Training Programs (FETPs) have expanded PHEM training, though coverage and integration remain uneven.^11^

Despite these and other important PHEM capacity-building developments on the continent, significant gaps remain. Rapidly evolving health threats and limited integration of new technologies such as genomic surveillance, geospatial analytics, climate analytics, artificial intelligence (AI), machine learning (ML) and other innovative digital health technologies may have rendered existing training frameworks and curricula insufficient to meet current and future workforce demands.^17,18^ Furthermore, Prevention of Sexual Exploitation, Abuse and Harassment (PRSEAH) during public health emergencies and the humanitarian-development-peace nexus, two relatively new phenomena that require specialised skill sets, may also not have been captured in existing trainings. Additionally, poor integration and fragmentation of training initiatives limit their effectiveness and sustainability, while many training programmes are insufficiently tailored to Africa’s diverse epidemiological and humanitarian contexts and PHEM competency needs.^15,19^

This scoping review therefore seeks to map the landscape of PHEM training programmes and curricula in Africa between 2000 and 2025, with the aim of identifying trends, strengths and persisting gaps. This is with a view to informing the development of more comprehensive, up-to-date, integrated and context-specific PHEM training programmes for the continent. We selected the 2000–2025 period for this scoping review as it captures a pivotal phase in the evolution of PHEM. The early 2000s saw the emergence and re-emergence of major outbreaks, including severe acute respiratory syndrome and pandemic influenza (H1N1), which prompted the development and strengthening of global health security frameworks such as the International Health Regulations (2005) and the Global Outbreak Alert and Response Network. Subsequent large-scale events, notably the 2014–2015 West Africa EVD outbreak and the COVID-19 pandemic, and the emergence of innovations such as AI/ML and emerging concepts such as the PRSEAH further shaped PHEM training priorities. Collectively, this 26-year timeframe provides a sufficiently comprehensive basis to examine the evolution of PHEM training programmes in Africa.

The review conceptualises PHEM and its training programmes from two inter-related perspectives. The first focuses on managing emergencies arising from infectious disease outbreaks such as EVD, mpox, cholera and yellow fever. The second concerns public health management in humanitarian crises, including armed conflict and natural disasters like floods and droughts. While overlapping, these contexts require distinct competencies. Outbreak-related PHEM emphasises surveillance, investigation, vaccination, infection prevention and control and risk communication, whereas humanitarian PHEM includes broader public health services and determinants such as water, sanitation and nutrition.

Against the backdrop of the foregoing, we guided this scoping review with the following questions:

1. What PHEM educational programmes and curricula have been developed or implemented in Africa since 2000?
2. What core competencies are included in these programmes and curricula?
3. What delivery methods are used (eg, short courses, certificated or uncertificated or formal accredited programmes offered by academic or training institutions)?
4. What evaluation methods have been used for these programmes?
5. What gaps and opportunities remain for improving PHEM capacity building in Africa?

## Methods

### Study design and setting

We conducted a scoping review to map and characterise existing training programmes and curricula for PHEM in all 54 countries of Africa between 2000 and 2025. Our approach was guided by the methodological framework of Arksey and O’Malley,^20^ as further refined by Levac *et al*,^21^ and reported in accordance with the Preferred Reporting Items for Systematic Reviews and Meta-Analyses extension for Scoping Reviews (PRISMA-ScR).^22^ The review focused exclusively on emergency training initiatives of public health importance relevant to Africa, covering both formal and informal educational initiatives led by governments, academic institutions, regional bodies and international partners. To enhance transparency and reduce the risk of bias, the review protocol was prospectively registered on the Open Science Framework at https://osf.io/prcu5/?view_only=c4940af218884778ac8e5061644c1a45.

### Information sources and search strategy

A comprehensive search strategy was designed to identify both peer-reviewed and grey literature. We searched the bibliographic databases of Ovid MEDLINE, PubMed, Scopus and CINAHL (accessed via the University of South Wales Online Library) on 3 September and 4 September 2025. To ensure the inclusion of non-indexed but relevant resources, we also searched organisational repositories and websites of WHO AFRO and Africa CDC, *African Journals Online* and Google Scholar (searched the first 15 pages) on 4 September 2025. The search strategy was built using Ovid MEDLINE with Boolean operators and later translated to the other databases. The search strategy combined four main concepts: *“Public Health Emergency Management”*, “*public health training”, “competency frameworks*” and “*Africa”*. A comprehensive search strategy was developed, incorporating synonyms and controlled vocabulary for each concept. The search strategy on Ovid MEDLINE combined controlled vocabulary (MeSH) and free-text terms, while the other databases used only free-text terms. Boolean operators (AND, OR), truncations and proximity searches were applied where appropriate to refine the search and maximise coverage. Full database-specific strategies are presented as online supplemental material 1.

### Eligibility criteria

We included studies and reports published between January 2000 and September 2025 that described the design, implementation or evaluation of PHEM training programmes or initiatives in Africa. Eligible sources encompassed peer-reviewed articles, technical reports, academic theses and organisational publications. We considered interventions that explicitly addressed PHEM competencies such as leadership, surveillance, risk communication, incident management, logistics and the integration of digital technologies. Exclusion criteria were applied to publications that lacked direct relevance to training or curricula, for example, general emergency response capacity papers that focused solely on clinical or biomedical training without a public health component, or systematic or scoping literature reviews, editorials, opinion pieces or studies conducted outside Africa.

#### Screening and data management

**Figure 1:**
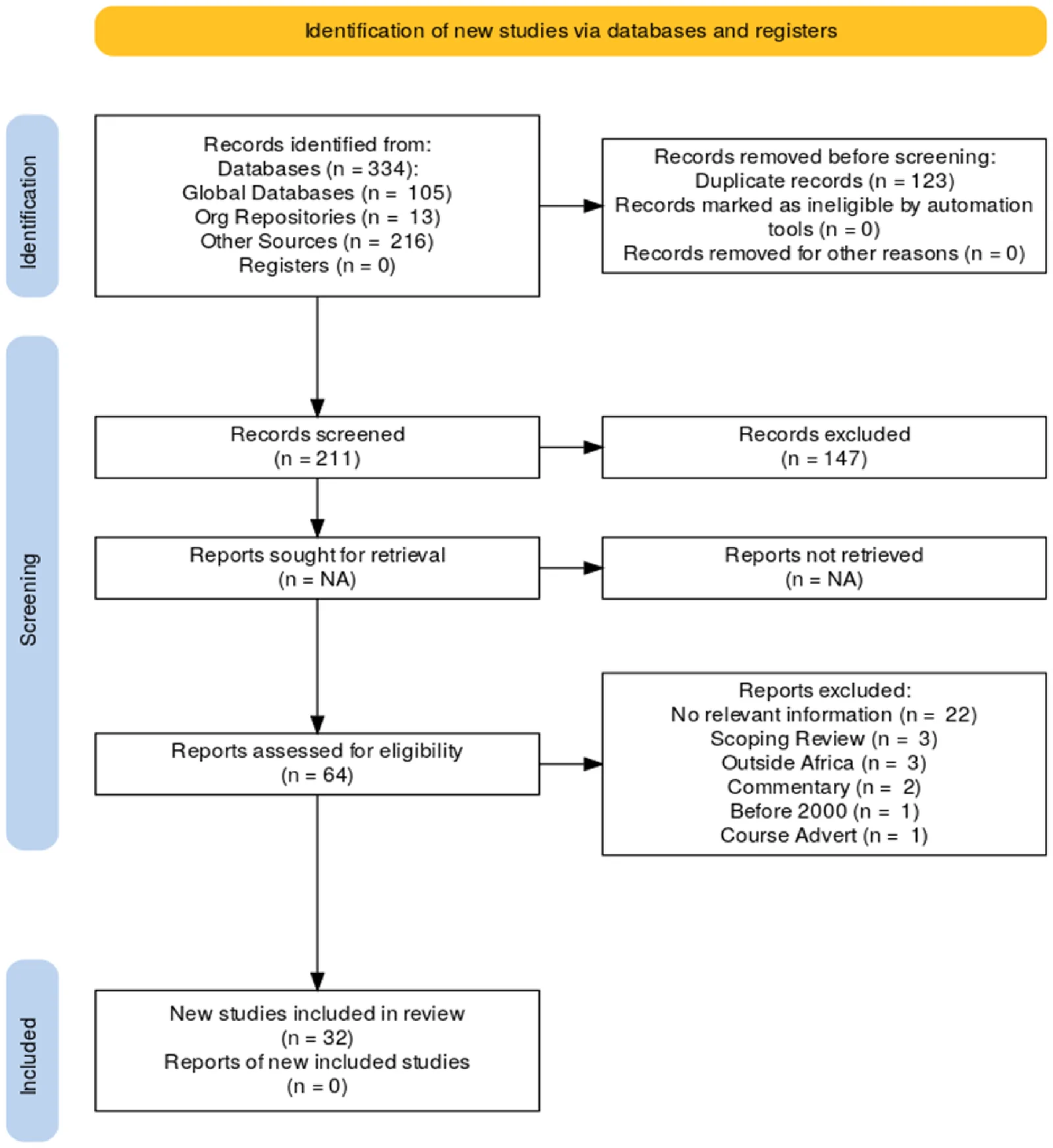
PRISMA-ScR flow diagram, highlighting the number of records identified, screened, excluded and included. PRISMA-ScR, Preferred Reporting Items for Systematic Reviews and Meta-Analyses extension for Scoping Reviews.

All search results were imported into Rayyan,^23^ an online tool for systematic and scoping reviews, which was used to remove duplicates, organise records, facilitate blinded screening and generate a PRISMA-ScR flow diagram. The screening process was carried out by two independent reviewers (OOO and RL) in two stages: (1) title and abstract screening to exclude clearly irrelevant records, followed by (2) full-text review to assess eligibility against the inclusion and exclusion criteria. Any disagreements at either stage were resolved through discussion, and, when necessary, a third reviewer served as an adjudicator. The overall selection process is summarised in a PRISMA-ScR flow diagram presented in figure 1, highlighting the number of records identified, screened, excluded and included.

#### Data extraction and charting

We developed and piloted a standardised data extraction form to ensure consistency and comparability across studies. For each included source, data were charted on bibliographic details (author, year, study title, design, location, source type, target audience and study focus), type of curriculum or programme, lead institution (eg, Ministry of Health, academic institution, Africa CDC, WHO, NGO), target audience (cadre of health workers and level of training), competencies addressed, delivery modality (eg, short course, fellowship, online, blended, in-person, accredited or non-accredited), duration of training, evaluation methods, reported outcomes and documented gaps and challenges. The tool was pilot tested on a subset of studies by RL and refined following discussion with OOO to improve clarity and accuracy. Final extraction was conducted by RL and verified independently by OOO to minimise bias and ensure completeness. The extracted data subsequently formed the basis for thematic coding and synthesis.

#### Data analysis and synthesis

The scoping review yielded mainly qualitative findings, which were analysed thematically, with RL conducting the initial coding and OOO reviewing codes for consistency. An inductive thematic analysis approach was used. Following data extraction, RL conducted a detailed review of all included studies and developed a preliminary coding framework based on recurrent concepts emerging from the data. Initial codes were generated around programme characteristics, competencies, delivery modalities, evaluation approaches, outcomes, challenges and opportunities for strengthening PHEM training. These codes were subsequently grouped into broader thematic categories aligned with the review objectives and research questions.

Recurring themes were identified around curriculum content, competencies, delivery modalities, evaluation strategies and challenges, and these were mapped against the review questions and situated within broader PHEM capacity-building frameworks. To enhance analytical rigour, OOO independently reviewed the coding framework, coded extracts and emerging themes for consistency. Areas of disagreement or ambiguity were discussed and resolved through consensus.

Themes were iteratively refined through team discussions to ensure conceptual clarity and robustness. Theme development was an iterative process involving repeated review of the extracted data to ensure that interpretations remained grounded in the original sources. To minimise interpretative bias, coding decisions, theme definitions and thematic groupings were discussed throughout the analytical process, and final themes were jointly agreed by RL and OOO before synthesis. The resulting themes informed the narrative synthesis and were mapped against broader PHEM capacity-building frameworks.

#### Ethical considerations

As this study relied exclusively on publicly available literature, ethical approval was not required. Nonetheless, the review was conducted in line with established principles of research integrity, including transparent reporting, accurate referencing and acknowledgement of all sources. Patients or the public were not involved in the design, conduct, reporting or dissemination plans of our research since it is a scoping review.

## Result

### Outcome of literature search and characteristics of the included studies

The outcome of the literature search on 3 and 4 September 2025 is presented in figure 1. In summary, the search identified 334 records from databases, repositories and other sources. After removing duplicates and screening titles and abstracts, 211 records were assessed, with 147 excluded. Of 64 full texts reviewed, 32 were excluded, leaving 32 studies included in the review.

The 32 studies that met the inclusion criteria were published between 2005 and 2025,^12,13,15,16,24–51^ with most (n=27) appearing after 2015.^12,13,15,16,25–31,33–37,39–46,48,50,51^ The studies employed a range of methodological approaches, with descriptive reports being the most common (n=13),^13,16,30,35,37,38,40–42,44,46,47,50^ followed by mixed methods designs (n=10),^12,15,24,25,28,29,31–33,46^ cross-sectional studies (n=4),^26,34,36,39^ pretest and post-test evaluations (n=2)^45,51^ and narrative literature review (n=1).^27^ The vast majority of the evidence was published in peer-reviewed journals (n=30), complemented by two grey literature sources.^43,49^

The training programmes targeted diverse groups within the public health workforce. The FETPs/FELTPs and their derivatives such as the frontline, intermediate and advanced courses were the most prominent. The studies addressed a range of focus areas, with FETPs/FETPs emerging as the backbone of PHEM workforce capacity strengthening across the continent.^24,25,30,37,38,40–42,44,45,47,48,51^ Evaluations of effectiveness and impact, particularly at the frontline level, were also prominent.^26,39,42,45,48,51^ A temporal shift was evident across the two decades of evidence. Earlier studies (2005–2014) largely documented the establishment of FETPs/FELTPs as foundational programmes for epidemiological capacity.^24,32,37,38,40,47^ In contrast, studies published after 2015 increasingly reflected diversification and innovation in training, with growing emphasis on frontline epidemiology, competency-based curricula and the integration of emerging themes such as One Health (OH), gender responsiveness, humanitarian crisis preparedness and digital tools.^13,15,16,33,35,43,49,50^ Detailed information about the study characteristics is presented in table 1 and online supplemental materials 2 and 3.

**Table 1:** Overview of the evidence base obtained from review (n=32)

Domain	Summary finding
Publication period	17 (53.1%) of the included studies were published between 2021 and 2025, indicating growing interest in PHEM workforce development in Africa.
Geographic coverage	Studies were identified across all African subregions, with East Africa contributing the largest share of the evidence base. Multicountry and regional initiatives were also frequently represented.
Study focus	Most studies focused on PHEM training programmes, curricula, competency frameworks, and workforce capacity development initiatives.
Training audience	Training programmes primarily targeted field epidemiologists, public health practitioners, emergency managers and multidisciplinary public health emergency response personnel.
Delivery approaches	A range of delivery approaches were reported, including in-person, blended, online and fellowship-based training models.
Competency areas	The most frequently addressed competencies included epidemiology, surveillance, outbreak investigation, emergency coordination, leadership, risk communication and One Health approaches.
Evaluation methods	Most studies reported descriptive, process or short-term training outcomes, while relatively few assessed longer-term workforce performance, emergency response capacity or programme impact.

Detailed characteristics of all included studies are presented in online supplemental materials 2 and 3.

PHEM, public health emergency management.

### Types of PHEM training programmes in Africa

The 32 included studies described a wide range of PHEM training initiatives ranging from short courses of a few days^28,32,35,36^ to long-term advanced fellowships lasting 24 months.^24,25,30,37,38,40,41,46,47^ Intermediate programmes typically spanned 3–6 months,^26,39,42,44,45,48,51^ while emerging models such as the Africa CDC PHEM fellowship were structured as intensive 6- to 11-week programmes.^15,16^ Longer academic pathways that awarded bachelor’s^27^ and master’s degrees^24,25,37,38,41,47^ in select programmes were also evident. The majority, however, issued certificates of completion, with fellowship programmes^15,16,30,34^ providing accredited recognition.

### Thematic areas and competencies covered in PHEM training programmes in Africa

Field epidemiology and surveillance emerged as the dominant training and technical area across reviewed programmes, reported in 17 (53%) of the 32 studies, comprising frontline, intermediate and advanced courses.^24–26,30,34,37–42,44–48,51^ Emergency preparedness and response was the next most frequent domain, cited in 10 (31%) studies, particularly through short courses and disaster management training.^12,13,15,16,27,28,32,36,49,50^ Leadership and management competencies appeared in 7 (22%) studies, typically within advanced fellowships and postgraduate-level programmes.^15,16,24,30,34,40,46,47^ OH and multisectoral competencies were highlighted in only 3 (9%) studies.^31,33,35^ Specialised technical skills such as infection prevention and control, safe burials, Geographic Information System (GIS), psychosocial support, humanitarian law and risk communication were also documented in only 6 (19%) studies.^28,29,32,36,43,49^ A few studies also emphasised the integration of gender-responsive approaches and intersectoral collaboration, particularly within OH frameworks.^31,33,35^ Table 2 provides more details on technical areas and competencies covered in the available PHEM training programmes in Africa.

**Table 2:** Competencies reported in PHEM training programmes in Africa by thematic area

Thematic area	Competencies
Introduction to Disaster Risk Management (DRM)	Research methods, grantsmanship and systems thinking Disaster risk management and emergency preparedness Safety and security in emergency settings
Operational effectiveness	Incident management and Public Health Emergency Operation Centre (PHEOC) operations Monitoring, evaluation and quality improvement
Effective leadership	Leadership, management and governance Programme and project management Field residency, mentorship and case studies
Preparedness and risk reduction	Risk communication and community engagement Legal, ethical and human rights considerations
Emergency response	Humanitarian response (Water, Sanitation and Hygiene (WASH), nutrition, protection, shelter) Paediatric and vulnerable groups’ disaster care
Postemergency health system recovery	Health systems and population recovery planning and implementation
One Health	One Health approaches (human-animal-environment interface) Environmental and ecosystem health
Gender & equity in health systems	Gender analysis and equity in epidemic response
Psychosocial and community support	Psychosocial support and mental healthcare
Diagnostics and laboratory systems	Public health laboratory methods and biosafety Quality systems and diagnostic procedures
Data management & analysis	Basic and advanced epidemiology Biostatistics and data analysis Public health surveillance Outbreak investigation and response
Supply chain & logistics	Logistics, supply chain and resource mobilisation
Digital health	Information technology, GIS and digital innovations
Public health communication/research and development	Communication, scientific writing and health reporting

PHEM, public health emergency management.

### Training method and evaluation strategies of PHEM training programmes in Africa

Most of the training programmes combined classroom-based instruction with fieldwork placements.^24,26,30,37,40,41,44–48,51^ Shorter initiatives relied more heavily on didactic workshops, simulations and case studies.^28,32,35,36^ More recent efforts increasingly adopted hybrid formats that blended online modules with in-person instruction,^13,15,16,31,49^ while several placed greater emphasis on peer learning, mentorship and applied projects to consolidate skills.^25,29,34,46^

Evaluation approaches varied considerably. Common strategies included pretests and post-tests to measure knowledge acquisition,^28,29,32,36,42,45,48,51^ while more advanced programmes incorporated portfolio reviews, thesis defences and accreditation processes, particularly in FELTPs and fellowship-level training.^24,30,34,40,41,46,47^ A few studies reported innovative methods, such as scorecard tools^41^, SMS-based reporting rate monitoring^29^ and real-time assessment of participants’ contributions to emergency responses.^46^

### Certification methods of PHEM training programmes in Africa

Certification pathways also differed markedly across programmes. Some were embedded in academic institutions, offering MSc degrees^24,25,34,37,38,40,41,47^ or BSc qualifications in longer courses.^27^ Structured fellowships, often led by Africa CDC or its partners, provided accredited certificates of completion.^15,16,30,34^ In contrast, the majority of short- and intermediate-level courses awarded only certificates of participation,^12,13,26,28,29,32,33,35,36,39,42–45,48–51^ with limited recognition for career progression or professional accreditation.

### Challenges associated with PHEM training programmes in Africa

Sustainability and funding constraints were the most frequently reported challenges, cited in 12 (38%) studies and often linked to donor dependence and weak government ownership.^13,16,24,25,30,34,37,40–42,47,50^ Human resource and retention challenges were noted in 9 (28%) studies, particularly high turnover rates of trained personnel, competing priorities and the difficulty of retaining fellows within national systems.^24,25,39–41,45,46,48,51^ Curriculum gaps and limited coverage were also highlighted in 9 (28%) studies, where training showed uneven emphasis on key competencies, underdeveloped laboratory tracks and limited evaluation mechanisms.^12,15,25,27,31,33,35,43,49^ Weak institutional integration was reported in 7 (22%) studies, especially poor linkages between FELTP/FETP graduates and ministries of health or universities.^16,26,30,38,40,47,50^ Finally, operational and contextual barriers appeared in 6 (19%) studies, encompassing political instability, language constraints, weak infrastructure, poor internet access and clinical workload pressures.^13,28,29,32,36,44^ Box 1 and online supplemental material 2 provide more detailed information on the challenges of PHEM training programmes in Africa.

Box 1 **Challenges associated with PHEM training programmes in Africa**

- Sustainability and reliance on donor funding.
- Limited government ownership and weak institutionalisation.
- Shortage of qualified mentors and supervisors.
- High staff turnover and weak retention pathways.
- Lack of standardised and accredited curricula.
- Weak integration into universities and health systems.
- Limited diagnostic capacity and laboratory infrastructure gaps.
- Language barriers and limited access to learning resources.
- Inconsistent mentorship and limited supervision at field sites.
- Poor transportation, logistics and field access.
- Limited access to PPE, testing kits and simulation resources.
- Understaffing and infrastructure constraints in facilities.
- Limited disaster training in older curricula.
- Low emphasis on gender, culture and social sciences.
- Overloaded medical curricula limiting integration of new topics.
- Political instability and coordination challenges.
- Limited monitoring, evaluation and career tracking mechanisms.
- Concerns about sustainability of short-term trainings.
- Limited research on effectiveness of training programmes.
- Lack of career pathways and progression for graduates.

PHEM, public health emergency management.

## Discussion

This study provides the first continent-wide review and analysis of PHEM training programmes in Africa. It highlights a divergence between current training approaches and the complex public health emergency landscape. While training initiatives have expanded in the past decade, they remain predominantly epidemic-focused and insufficiently adapted to complex emergencies, including humanitarian crises. The study also reveals challenges and structural weaknesses in current PHEM training programmes and raises the issue of limited emphasis on key cross-cutting areas such as OH, intersectoral collaboration and gender-sensitive approaches to PHEM. Few, if any, addressed contemporary themes such as PRSEAH, the application of innovative technologies to PHEM and the humanitarian-development-peace nexus.

The predominance of epidemic management-oriented courses is unsurprising, as epidemics remain the most frequent public health emergencies in Africa. This focus intensified after the 2014–2016 EVD epidemic in West Africa, which exposed the severe shortage of a well-trained PHEM health workforce. Our observations and available evidence suggest that clear differentiation between public health emergencies and public health management in humanitarian crises is relatively recent.^52–54^ Consequently, many older PHEM training programmes may not have incorporated this distinction.^52^ Another plausible explanation for this finding is the long-standing and substantial funding support (mainly from the USA government) targeted at strengthening epidemic management-focused FETP/FELTP programmes across Africa.^55^ Nevertheless, the growing frequency of humanitarian crises highlights the need for broader and integrated PHEM training programmes^56^ that encompass both epidemic response and public health management in complex emergencies. Such training will equip health professionals with wide-ranging competencies to function effectively across diverse emergency contexts. Furthermore, opportunities for specialisation and subspecialisation within PHEM should be developed to build a sustainable pool of expert practitioners on the continent.^12^

The limited emphasis on emerging areas and competencies such as OH, PRSEAH, the humanitarian-development-peace nexus and the application of innovative technologies such as AI and ML may be attributed to their novelty and limited understanding of their effective application in PHEM. For example, the issue of PRSEAH gained prominence only during the 2017–2018 EVD outbreaks in the Democratic Republic of Congo, when serious incidents of sexual exploitation of affected populations were reported.^57^ Similarly, the use of AI and ML in public health has become popular only in recent years.^18^ Although several studies have documented the potential role of these technologies in epidemic management globally,^58,59^ their implementation in Africa remains limited. Consequently, there are few lessons learnt or practical experiences on which to build core competencies. Furthermore, the scarcity of skilled trainers in these emerging areas likely contributes to their limited inclusion in existing PHEM training programmes.

Attainment of PHEM competency requires the acquisition of relevant knowledge, technical skills and appropriate professional behaviours in emergency contexts. Therefore, the current emphasis on evaluating only knowledge and skills among PHEM trainees warrants further investigation. This limitation may stem from the difficulty of assessing behavioural change in classroom or simulated settings. While knowledge and skills can be measured through pretests and post-tests or practical assessments such as theses and manuscript development, the evaluation of behavioural change requires longitudinal observation, ideally in real-life emergency situations, which is often impractical. Previous evaluations of PHEM training programmes, such as those of the FETP, have focused primarily on training outcomes and programmatic impact, not on individual behavioural change.^60^ Although Magnaye (2025)^61^ has proposed the use of reflective and analytical assessment methods to bridge the gap between theory and practice in PHEM training,^61^ further research is needed to develop effective approaches for assessing the behavioural change component of PHEM competency within simulated or academic settings in the African context.

The instructional methods employed by most African PHEM training programmes are consistent with those used globally.^61,62^ However, recent literature highlights additional modalities, such as AI-assisted learning, telemedicine platforms, online games and interactive quizzes as innovative tools for delivering PHEM training.^62^ These methods present new opportunities that could be adapted to improve instructional quality and learner engagement across the current and future African PHEM training courses.

A further limitation identified is the lack of formal certification for most short- and intermediate-level PHEM courses. This restricts recognition for career advancement and professional accreditation, which may be a disincentive among trainees. Addressing this challenge requires a structured certification framework. One option involves awarding recognised certificates for intermediate courses, alongside a stepwise approach that allows learners to progress from short to intermediate and advanced levels. Such a framework would strengthen career pathways while promoting continuous professional development in PHEM across the continent.

The challenges to PHEM training programmes identified in this review are consistent with those reported in other contexts.^61^ These challenges represent major barriers to scaling up PHEM training across Africa and call for context-specific, locally driven solutions. One potential approach is the integration of PHEM training into existing medical and public health education curricula. Such integration would expand trainee coverage, reduce financial barriers and enhance sustainability. Incorporating PHEM training costs into national health budgets, as well as into the annual operational plans of regional and national public health institutions, could further address funding and sustainability constraints. Poor retention and high turnover of trained personnel could be mitigated through bonding mechanisms that require trainees to serve for a defined period within sponsoring or training institutions. However, such bonding mechanisms should not prevent the public good benefits of PHEM training. Furthermore, curriculum gaps and uneven emphasis on key competencies could be addressed through the development of a continent-wide PHEM training core competency framework. This framework should define essential competencies for various cadres of the health workforce across basic, intermediate and advanced levels.

### Implications for research, policy and practice

This scoping review identifies several critical evidence gaps that warrant further investigation and have important implications across the research–policy–practice continuum of PHEM training in Africa.

From a research perspective, there is a need for robust empirical studies to define, standardise and validate emerging PHEM competencies across different health workforce cadres, considering the evolving nature of public health emergencies and humanitarian contexts on the continent. In addition, methodological advancements are required to develop and validate evaluation frameworks capable of rigorously assessing the behavioural and applied dimensions of PHEM competencies, which remain insufficiently captured in existing training models. Furthermore, future research should systematically examine the role of digital health innovations, including e-learning platforms, simulation technologies and data-driven learning systems, in optimising both the delivery and evaluation of PHEM training. Generating evidence on the effectiveness, scalability and contextual applicability of such technologies will be critical for informing next-generation training approaches.

From a policy perspective, the findings highlight a notable absence of comprehensive, integrated national frameworks to guide the design, implementation and evaluation of PHEM training programmes. This underscores the need for the development of harmonised regional policy frameworks that can be adapted to country-specific contexts. Such policies should clearly delineate competencies required for managing public health emergencies and humanitarian crises, while promoting their integration within a unified competency-based framework. In addition, policy instruments should incorporate guidance on embedding cross-cutting priorities such as the humanitarian-development-peace nexus, emerging technological innovations and robust monitoring and evaluation systems, particularly those addressing behavioural competencies into PHEM training. Sustainable financing mechanisms must also be prioritised within such policy frameworks to ensure equitable access, continuity and long-term sustainability of PHEM training programmes across Africa.

At the level of practice, the findings emphasise the importance of developing and implementing evidence-based, competency-driven PHEM curricula that are responsive to contextual realities. Practitioners and training institutions should adopt innovative and adaptive delivery modalities to address identified systemic and operational challenges. Equally important is the establishment of structured mechanisms to facilitate the translation of research evidence into policy formulation and programmatic action. Strengthening these linkages will be essential for ensuring that PHEM training initiatives are not only scientifically grounded but also effectively implemented, thereby enhancing public health emergency preparedness and response capacity across the region.

### Study limitations

The findings of this study should be interpreted considering the inherent limitations of scoping review methodology. First, the review did not include a formal assessment of the methodological quality or risk of bias in the studies included. Consequently, translation of the findings into policy or practice should be approached with caution and contextual sensitivity. Second, the restriction of the search to a defined period and geographic area may have led to the omission of relevant evidence, examples or lessons from other regions. As such, the findings and recommendations may primarily be applicable to the African context. Third, the exclusion of non-English publications could have resulted in exclusion of evidence from such publications.

Finally, the findings of this scoping review should also be interpreted in light of several methodological limitations, including potential publication bias, the likely under-representation of unpublished training initiatives and the predominance of descriptive programme reports with limited outcome evaluation. These factors may have influenced the robustness of the findings and, consequently, the strength of the study’s recommendations.

To mitigate these limitations, a comprehensive and systematic search strategy was employed, encompassing both peer-reviewed and grey literature. In addition, rigorous screening and review procedures were applied to enhance the quality, relevance and completeness of data extraction and to support a robust synthesis of the available evidence.

## Conclusion

A competent public health workforce is fundamental to ensuring timely and effective responses to the increasing burden of public health emergencies and humanitarian crises in Africa. This review examined existing PHEM training programmes, identifying several strengths and opportunities as well as gaps and implementation challenges on the continent. The findings showed that, although multiple programmes exist, few comprehensively address both epidemic management and public health management within complex emergency settings. Additionally, emerging domains such as PRSEAH, the OH approach, use of innovative digital health technologies and the humanitarian-development-peace nexus are seldom integrated into current PHEM curricula in Africa. Persistent barriers, including inadequate funding, limited sustainability mechanisms and poor retention of trained personnel, further constrain the effectiveness and scalability of these programmes.

Based on the study findings and the associated limitations, we propose six key recommendations to strengthen PHEM training initiatives in Africa.

First, there is a need for the continuous mapping and characterisation of existing PHEM training programmes across the continent to generate a comprehensive evidence base. Such efforts would inform the strategic development and expansion of additional short-, medium- and long-term training initiatives that integrate competencies for both epidemic response and the management of public health functions during complex emergencies. Such programmes will equip graduates to function effectively across diverse emergency contexts.

Second, considering evolving public health threats, existing PHEM training curricula should be periodically reviewed and, where necessary, expanded to incorporate emerging priority areas and competencies. Such areas may include PRSEAH, OH approaches, intersectoral collaboration, gender-responsive programming, the humanitarian-development-peace nexus and the application of innovative technologies such as AI and ML.

Third, since competency encompasses knowledge, skills and behavioural change, efforts should be made to ensure that PHEM training evaluation frameworks include all three domains as much as practicable. The inclusion of behavioural change assessment tools such as simulated or observed emergency scenarios would complement existing pretests and post-tests and thesis evaluations.

Fourth, sustainable financing mechanisms for PHEM training programmes should be developed. In this regard, the integration of PHEM training into national and institutional budgets would help ensure continuity and reduce dependence on external funding.

Fifth, building on the existing evidence on PHEM training, generic core competency frameworks for short, intermediate and advanced-level courses should be updated for different cadres of the African public health workforce. Additionally, establishing a pool of certified PHEM trainers would further promote standardisation and quality across the continent.

Lastly, further research is essential to identify emerging competencies, develop innovative course evaluation and certification methods and design strategies for long-term programme sustainability.

Together, we believe that these actions will help build a resilient, well-trained public health workforce capable of safeguarding Africa’s populations against current and future public health emergencies.

## Supplementary material

10.1136/bmjph-2026-004980 online supplemental file 1

10.1136/bmjph-2026-004980 online supplemental file 2

10.1136/bmjph-2026-004980 online supplemental file 3

## Data Availability

All data relevant to the study are included in the article or uploaded as supplementary information.
