# Supplementary material for: Scoping review of public health emergency management training programmes and curricula in Africa (2000–2025): implications for policy, practice and research
Source: BMJ Public Health. 2026 Sep 21;4(Suppl 1):e004980. doi: 10.1136/bmjph-2026-004980 (PMC13599839; doi:10.1136/bmjph-2026-004980)
Supplement: Supplementary file 1 [file bmjph-4-Suppl_1-s001.pdf]

S/No.	Key Concept	MEDLINE/ SEARCH TERMS	RESULTS
1	Public Health Emergency Management	exp Emergency Preparedness/	11,314
2		exp Disaster Planning/	20,049
3		exp Disaster Medicine/	24,159
4		exp Relief Work/	13,578
5		exp Emergency Medical Services/	374,078
6		exp Communicable Disease Control/	102,534
7		exp Public Health Administration/	1,858,435
8		"public health emergency management*" OR PHEM* OR "emergency preparedness*" OR "emergency response*" OR "incident management system*" OR "incident command system*" OR "disaster management*" OR "disaster response*" OR "health emergency*" OR "humanitarian emergency*" OR "outbreak response*" OR "epidemic control*" OR "pandemic preparedness*"	42,666
**9**		**1 or 2 or 3 or 4 or 5 or 6 or 7 or 8**	**42,669**
10	Public Health Training	exp Education/	38
11		exp Education, Public Health Professional/	314,998
12		exp Education, Continuing/	95,482
13		exp Education, Medical, Graduate/	420,164
14		exp Training Support/	126,643
15		exp Curriculum/	342
16		exp Teaching/	8
17		exp Internship and Residency/	1
18		"Public Health Training" OR "training" OR "training program*" OR "education program*" OR "curriculum" OR "course content" OR "capacity building" OR "academic training" OR "professional development" OR "certificate program*" OR "short course*" OR "public health education" OR "learning module*" OR "instructional design" OR "Field	1,024,572

		Epidemiology Training Program" OR "FETP" OR "applied epidemiology training" OR "epidemiology fellowship"	
**19**		**10 or 11 or 12 or 13 or 14 or 15 or 16 or 17 or 18**	**1,024,572**
20	Competency Frameworks	exp Competency-Based Education/	33,636
21		exp Professional Competence/	50,786
22		exp Clinical Competence/	137,933
23		exp Health Personnel/education/	347,887
24		exp Learning/	48
25		competenc* OR "core competenc*" OR skills adj3 development OR "learning outcome*" OR "competency-based education*" OR "public health skills*" OR "technical skills*" OR "knowledge area*" OR "performance indicator*"	281,702
**26**		**20 or 21 or 22 or 23 or 24 or 25**	**281,702**
27	Africa	exp Africa South of the Sahara/	19,849
28		exp Africa, Central/	29,674
29		exp Africa, Southern/	23,643
30		exp Africa, Western/	34,484
31		exp Africa, Eastern/	21,338
32		exp Africa, Northern/	18,499
33		Africa* OR "Sub-Saharan Africa*" OR "West* Africa*" OR "East* Africa*" OR "Southern Africa*" OR "North* Africa*" OR Algeria* OR Angola* OR Benin* OR Botswana* OR "Burkina Faso*" OR Burundi* OR Cameroon* OR "Cape Verde*" OR "Central African Republic*" OR Chad* OR Comoros* OR Congo* OR "Democratic Republic of Congo*" OR DRC OR Djibouti* OR Egypt* OR "Equatorial Guinea*" OR Eritrea* OR Eswatini* OR Ethiopia* OR Gabon* OR Gambia* OR Ghana* OR Guinea* OR "Guinea-Bissau*" OR "Ivory Coast*" OR "Côte d'Ivoire*" OR Kenya* OR Lesotho* OR Liberia* OR Libya* OR Madagascar* OR Malawi* OR Mali* OR Mauritania* OR Mauritius* OR Morocco* OR Mozambique* OR Namibia* OR Niger* OR Nigeria* OR Rwanda* OR "Sao Tome and Principe*" OR Senegal* OR Seychelles* OR "Sierra	2,177,954

		Leone*" OR Somalia* OR "South* Africa*" OR "South* Sudan*" OR Sudan* OR Tanzania* OR Togo* OR Tunisia* OR Uganda* OR Zambia* OR Zimbabwe*	
**34**		**27 or 28 or 29 or 30 or 31 or 32 or 33**	**2,177,954**
**35**		**9 AND 19 AND 26 AND 34**	**65**
**36**	**Limits**	**Full text/English Language/Custom date 01/01/2000-04/09/2025**	**25**
**S.No.**	**Key Concept**	**SCOPUS SEARCH TERMS**	**RESULTS**
1	Public Health Emergency Management	TITLE-ABS-KEY("public health emergency management*" OR PHEM* OR "emergency preparedness*" OR "emergency response*" OR "incident management system*" OR "incident command system*" OR "disaster management*" OR "disaster response*" OR "health emergency*" OR "humanitarian emergency*" OR "outbreak response*" OR "epidemic control*" OR "pandemic preparedness*")	105,565
2	Public Health Training	TITLE-ABS-KEY("Public Health Training" OR training OR "training program*" OR "education program*" OR curriculum OR "course content" OR "capacity building" OR "academic training" OR "professional development" OR "certificate program*" OR "short course*" OR "public health education" OR "learning module*" OR "instructional design" OR "Field Epidemiology Training Program" OR FETP OR "applied epidemiology training" OR "epidemiology fellowship")	2,550,748
3	Competency Frameworks	TITLE-ABS-KEY(competenc* OR "core competenc*" OR skills W/3 development OR "learning outcome*" OR "competency-based education*" OR "public health skills*" OR "technical skills*" OR "knowledge area*" OR "performance indicator*")	94,924
4	Africa	TITLE-ABS-KEY(Africa* OR "Sub-Saharan Africa*" OR "West* Africa*" OR "East* Africa*" OR "Southern Africa*" OR "North* Africa*" OR Algeria* OR Angola* OR Benin* OR Botswana* OR "Burkina Faso*" OR Burundi* OR Cameroon* OR "Cape Verde*" OR "Central African Republic*" OR Chad* OR Comoros* OR Congo* OR "Democratic Republic of Congo*" OR DRC OR Djibouti* OR Egypt* OR "Equatorial Guinea*" OR Eritrea* OR Eswatini* OR Ethiopia* OR Gabon* OR Gambia* OR Ghana* OR Guinea* OR "Guinea-Bissau*" OR "Ivory Coast*" OR "Côte d'Ivoire*" OR Kenya* OR Lesotho*	3,234,684

		OR Liberia* OR Libya* OR Madagascar* OR Malawi* OR Mali* OR Mauritania* OR Mauritius* OR Morocco* OR Mozambique* OR Namibia* OR Niger* OR Nigeria* OR Rwanda* OR "Sao Tome and Principe*" OR Senegal* OR Seychelles* OR "Sierra Leone*" OR Somalia* OR "South* Africa*" OR "South* Sudan*" OR Sudan* OR Tanzania* OR Togo* OR Tunisia* OR Uganda* OR Zambia* OR Zimbabwe*)	
5		**1 AND 2 AND 3 AND 4**	12
**6**	**Limits**	**English Language/2000-2025**	**12**
**S.No.**	**Key Concept**	**CINAHL SEARCH TERMS**	**RESULTS**
1	Public Health Emergency Management	TI"public health N5 emergency N5 management*" OR PHEM* OR "emergency preparedness*" OR "emergency response*" OR "incident N3 management system*" OR "incident N3 command system*" OR "disaster management*" OR "disaster response*" OR "health emergency*" OR "humanitarian emergency*" OR "outbreak response*" OR "epidemic control*" OR "pandemic preparedness*")<br><br>OR<br><br>AB ("public health N5 emergency N5 management*" OR PHEM* OR "emergency preparedness*" OR "emergency response*" OR "incident N3 management system*" OR "incident N3 command system*" OR "disaster management*" OR "disaster response*" OR "health emergency*" OR "humanitarian emergency*" OR "outbreak response*" OR "epidemic control*" OR "pandemic preparedness*"	18,270
2	Public Health Training	TI ("public health N3 training" OR training OR "training program*" OR "education program*" OR curriculum OR "course content" OR "capacity building" OR "academic training" OR "professional development" OR "certificate program*" OR "short course*" OR "public health N3 education" OR "learning module*" OR "instructional design" OR "Field Epidemiology Training Program" OR FETP OR "applied epidemiology N3 training" OR "epidemiology fellowship")	412,596

		OR<br><br>AB ("public health N3 training" OR training OR "training program*" OR "education program*" OR curriculum OR "course content" OR "capacity building" OR "academic training" OR "professional development" OR "certificate program*" OR "short course*" OR "public health N3 education" OR "learning module*" OR "instructional design" OR "Field Epidemiology Training Program" OR FETP OR "applied epidemiology N3 training" OR "epidemiology fellowship")	
3	Competency Frameworks	TI (competenc* OR "core competenc*" OR skills N3 development OR "learning outcome*" OR "competency-based education*" OR "public health skills*" OR "technical skills*" OR "knowledge area*" OR "performance indicator*")<br><br>OR<br><br>AB (competenc* OR "core competenc*" OR skills N3 development OR "learning outcome*" OR "competency-based education*" OR "public health skills*" OR "technical skills*" OR "knowledge area*" OR "performance indicator*")	148,326
**4**	**Africa**	TI (Africa* OR "Sub-Saharan Africa*" OR "West* Africa*" OR "East* Africa*" OR "Southern Africa*" OR "North* Africa*" OR Algeria* OR Angola* OR Benin* OR Botswana* OR "Burkina Faso*" OR Burundi* OR Cameroon* OR "Cape Verde*" OR "Central African Republic*" OR Chad* OR Comoros* OR Congo* OR "Democratic Republic of Congo*" OR DRC OR Djibouti* OR Egypt* OR "Equatorial Guinea*" OR Eritrea* OR Eswatini* OR Ethiopia* OR Gabon* OR Gambia* OR Ghana* OR Guinea* OR "Guinea-Bissau*" OR "Ivory Coast*" OR "Côte d'Ivoire*" OR Kenya* OR Lesotho* OR Liberia* OR Libya* OR Madagascar* OR Malawi* OR Mali* OR Mauritania* OR Mauritius* OR Morocco* OR Mozambique* OR Namibia* OR Niger* OR Nigeria* OR Rwanda* OR "Sao Tome and Principe*" OR Senegal* OR Seychelles* OR "Sierra Leone*" OR Somalia* OR "South* Africa*" OR "South* Sudan*" OR Sudan* OR Tanzania* OR Togo* OR Tunisia* OR Uganda* OR Zambia* OR Zimbabwe*)	297,612

		OR<br><br>AB (Africa* OR "Sub-Saharan Africa*" OR "West* Africa*" OR "East* Africa*" OR "Southern Africa*" OR "North* Africa*" OR Algeria* OR Angola* OR Benin* OR Botswana* OR "Burkina Faso*" OR Burundi* OR Cameroon* OR "Cape Verde*" OR "Central African Republic*" OR Chad* OR Comoros* OR Congo* OR "Democratic Republic of Congo*" OR DRC OR Djibouti* OR Egypt* OR "Equatorial Guinea*" OR Eritrea* OR Eswatini* OR Ethiopia* OR Gabon* OR Gambia* OR Ghana* OR Guinea* OR "Guinea-Bissau*" OR "Ivory Coast*" OR "Côte d'Ivoire*" OR Kenya* OR Lesotho* OR Liberia* OR Libya* OR Madagascar* OR Malawi* OR Mali* OR Mauritania* OR Mauritius* OR Morocco* OR Mozambique* OR Namibia* OR Niger* OR Nigeria* OR Rwanda* OR "Sao Tome and Principe*" OR Senegal* OR Seychelles* OR "Sierra Leone*" OR Somalia* OR "South* Africa*" OR "South* Sudan*" OR Sudan* OR Tanzania* OR Togo* OR Tunisia* OR Uganda* OR Zambia* OR Zimbabwe*)	
**5**		**1 and 2 and 3 and 4**	**15**
**6**	**Limits**	**English Language/Full text/Published 2000-2025**	**9**
**S.No.**	**Key Concept**	**PubMed SEARCH TERMS**	**RESULTS**
1	Public Health Emergency Management	("public health emergency management*"[All Fields] OR "phem*"[All Fields] OR "emergency preparedness*"[All Fields] OR "emergency response*"[All Fields] OR "incident management system*"[All Fields] OR "incident command system*"[All Fields] OR "disaster management*"[All Fields] OR "disaster response*"[All Fields] OR "health emergency*"[All Fields] OR "humanitarian emergency*"[All Fields] OR "outbreak response*"[All Fields] OR "epidemic control*"[All Fields] OR "pandemic preparedness*"[All Fields]) AND (2000/1/1:2025/9/3[pdat])	32,705
2	Public Health Training	("Public Health Training"[All Fields] OR "training"[All Fields] OR "training program*"[All Fields] OR "education program*"[All Fields] OR "curriculum"[All Fields] OR "course content"[All Fields] OR "capacity building"[All Fields] OR "academic training"[All Fields] OR "professional development"[All Fields] OR "certificate program*"[All Fields] OR "short course*"[All Fields] OR "public health education"[All Fields] OR "learning module*"[All Fields] OR "instructional design"[All Fields] OR "Field Epidemiology Training Program"[All Fields] OR "FETP"[All Fields] OR	869,681

		"applied epidemiology training"[All Fields] OR "epidemiology fellowship"[All Fields]) AND (2000/1/1:2025/9/3[pdat])	
3	Competency Frameworks	("competenc*"[All Fields] OR "core competenc*"[All Fields] OR (("skill"[All Fields] OR "skilled"[All Fields] OR "skillful"[All Fields] OR "skillfulness"[All Fields] OR "skills"[All Fields]) AND "adj3"[All Fields] AND ("develop"[All Fields] OR "develope"[All Fields] OR "developed"[All Fields] OR "developer"[All Fields] OR "developer s"[All Fields] OR "developers"[All Fields] OR "developing"[All Fields] OR "developments"[All Fields] OR "develops"[All Fields] OR "growth and development"[MeSH Subheading] OR ("growth"[All Fields] AND "development"[All Fields]) OR "growth and development"[All Fields] OR "development"[All Fields])) OR "learning outcome*"[All Fields] OR "competency based education*"[All Fields] OR "public health skills*"[All Fields] OR "technical skills*"[All Fields] OR "knowledge area*"[All Fields] OR "performance indicator*"[All Fields]) AND (2000/1/1:2025/9/3[pdat])	240,460
**4**	Africa	("africa*"[All Fields] OR "sub saharan africa*"[All Fields] OR "west* africa*"[All Fields] OR "east* africa*"[All Fields] OR "southern africa*"[All Fields] OR "north* africa*"[All Fields] OR "algeria*"[All Fields] OR "angola*"[All Fields] OR "benin*"[All Fields] OR "botswana*"[All Fields] OR "burkina faso*"[All Fields] OR "burundi*"[All Fields] OR "cameroon*"[All Fields] OR "cape verde*"[All Fields] OR "central african republic*"[All Fields] OR "chad*"[All Fields] OR "comoros*"[All Fields] OR "congo*"[All Fields] OR "democratic republic of congo*"[All Fields] OR "DRC"[All Fields] OR "djibouti*"[All Fields] OR "egypt*"[All Fields] OR "equatorial guinea*"[All Fields] OR "eritrea*"[All Fields] OR "eswatini*"[All Fields] OR "ethiopia*"[All Fields] OR "gabon*"[All Fields] OR "gambia*"[All Fields] OR "ghana*"[All Fields] OR "guinea*"[All Fields] OR "guinea bissau*"[All Fields] OR "ivory coast*"[All Fields] OR "cote d ivoire*"[All Fields] OR "kenya*"[All Fields] OR "lesotho*"[All Fields] OR "liberia*"[All Fields] OR "libya*"[All Fields] OR "madagascar*"[All Fields] OR "malawi*"[All Fields] OR "mali*"[All Fields] OR "mauritania*"[All Fields] OR "mauritius*"[All Fields] OR "morocco*"[All Fields] OR "mozambique*"[All Fields] OR "namibia*"[All Fields] OR "niger*"[All Fields] OR "nigeria*"[All Fields] OR "rwanda*"[All Fields] OR "sao tome and principe*"[All Fields] OR "senegal*"[All Fields] OR "seychelles*"[All Fields] OR "sierra leone*"[All Fields] OR "somalia*"[All Fields] OR "south* africa*"[All Fields] OR "south* sudan*"[All Fields] OR "sudan*"[All Fields] OR "tanzania*"[All Fields] OR "togo*"[All Fields] OR "tunisia*"[All Fields] OR "uganda*"[All Fields] OR	1,620,161

		"zambia*"[All Fields] OR "zimbabwe*"[All Fields]) AND (2000/1/1:2025/9/3[pdat])	
**5**		**1 and 2 and 3 and 4**	**59**
**S.No.**	**Key Concept**	**African Journals Online SEARCH TERMS**	**RESULTS**
1	Public Health Emergency Management<br><br>Public Health Training<br><br>Competency Frameworks	public health OR public health emergency OR emergency management OR outbreak management OR disease outbreak management AND curriculum OR curricula OR training OR skills OR competence OR competencies OR field epidemiology training programme OR feltp	**66**
**S.No.**	**Key Concept**	**Google Scholar SEARCH TERMS**	**RESULTS**
**1**	Public Health Emergency Management<br><br>Public Health Training<br><br>Competency Frameworks<br><br>Africa	Public Health Emergency Management, PHEM, Public Health Training, Disaster Response, Competency Frameworks, Africa, Sub-Saharan Africa, East Africa, West Africa, Central Africa, Southern Africa	**150**
