# Supplementary material for: Scoping review of public health emergency management training programmes and curricula in Africa (2000–2025): implications for policy, practice and research
Source: BMJ Public Health. 2026 Sep 21;4(Suppl 1):e004980. doi: 10.1136/bmjph-2026-004980 (PMC13599839; doi:10.1136/bmjph-2026-004980)
Supplement: Supplementary file 2 [file bmjph-4-Suppl_1-s002.pdf]

S/N	Study	Programme Name	Sub-category	Lead Agency	Duration	Competencies Covered	Delivery Method	Evaluation Approach	Certification	Challenges
1	Jima et al	FELTP	FELTP Advanced	AAU-SPH; FMOH, EHNRI, EPHA, CDC, AFENET	24 months	-Basic Epidemiology and Biostatistics<br>-Field Epidemiology<br>-Public Health Surveillance<br>-Public Health Laboratory Methods and Biosafety<br>-Communication and Scientific Writing<br>-Computer Applications in Public Health<br>-Management and Leadership<br>-Advanced Epidemiology and Epidemiology of Priority Health Problems in Ethiopia<br>-Disaster Management<br>-Field Residency I and II<br>-Projects I and II	-Classroom based<br>-Field-based	-Pre/post assessments<br>-Field assignments<br>-Surveillance system evaluations<br>-Outbreak investigations<br>-Abstracts and manuscript submissions<br>-Oral and poster presentations at scientific conferences	Certificated	-Limited mentoring and supervision at regional field bases<br>-Communication and information access barriers<br>-Lack of transportation for fieldwork and investigations
2	Gatei et al	FELTP	FELTP-L Track	CDC, AFENET & MOH	24 months	- Epidemiology<br>- Disease surveillance<br>- Outbreak response<br>- Laboratory management<br>- Quality systems<br>- Diagnostic procedures<br>- Policy and leadership	- Competency-based training<br>- Field assignments	- Surveys and questionnaires to FELTP directors<br>- CDC internal assessments<br>- Career tracking of graduates	Certificated	- Lack of standardized curriculum<br>- Structural issues and unclear purpose of L-Track<br>- Limited number of applicants<br>- Funding constraints (e.g., reduced support from PEPFAR, PMI)<br>- Difficulty integrating L-Track with FETP<br>- Shortage of mentors and training facilities<br>- Career drift away from core laboratory services
3	Kebebew et al	FETP	FETP–Frontline	EPHI, CDC, WHO	3 months	- Disease surveillance<br>- Outbreak investigation<br>- Data analysis and interpretation<br>- Monitoring and evaluation<br>- Scientific communication<br>- Use of Microsoft Excel, Word, PowerPoint<br>- SWOT analysis	-Classroom-based<br>- Fieldwork assignments<br>- Practical group exercises	-Structured questionnaire and observation checklist<br>- Comparison of trained vs. untrained officers	Certificated	- Trained officers had more prior training and experience, which may confound results<br>- No analysis of dropout or staff turnover<br>- Educational disparities between trained and

S/N	Study	Programme Name	Sub-category	Lead Agency	Duration	Competencies Covered	Delivery Method	Evaluation Approach	Certification	Challenges
						- Case investigation<br>- Supportive supervision<br>- Epidemiologic bulletin preparation	- On-the-job training			untrained groups<br>- Some surveillance components showed no significant difference between groups
4	Mbola et al	Disaster Management Training for Environmental Health Practitioners	-	TUT, CPUT, AAEH	-48 months (long course)<br>-2-6 days (short-course)	- Disaster risk reduction<br>- Emergency preparedness and response<br>- Community assessment<br>- Vulnerability mapping<br>- Communication and public awareness<br>- Hazard identification<br>- Disaster response planning<br>- Environmental health surveillance and control during disasters	- classroom based<br>-On-the-job training<br>- Workshops and seminars	- Global comparison of training efforts and gaps	-Long course, certificated<br>-Short course not certificated	- Lack of disaster management training in older EH curricula<br>- Limited published training in low- and middle-income countries<br>- Ambiguity in EHP roles during disasters<br>- Scarcity of research on EH disaster training<br>- Need for curriculum review and collaboration between academia and field practitioners
5	Siouris et al	COVID-19 Training Initiative for Frontline Health Workers	-	ICAP, RTSL, MOHs	1 to 8 days	- COVID-19 origins and transmission<br>- IPC principles and practices<br>- PPE usage<br>- Patient screening and triage<br>- Maintenance of essential services<br>- Effective communication and community engagement<br>- Use of IPC checklists<br>- Reporting and referral protocols	- Classroom based/online	- Pre- and post-test assessments<br>- Online post-training survey	Not certificated	- Limited access to PPE and testing kits<br>- Understaffing and infrastructure gaps<br>- Variation in training modalities and content across countries<br>- No direct measurement of behaviour change or facility-level IPC compliance<br>- Need for refresher and simulation-based training, especially for non-clinical staff

S/N	Study	Programme Name	Sub-category	Lead Agency	Duration	Competencies Covered	Delivery Method	Evaluation Approach	Certification	Challenges
6	Cáceres et al	STEP	-	CDC, MOHs, TEPHINET, AFENET, WHO	5 weeks	- Integrated Disease Surveillance and Response<br>- Ebola case investigation and contact tracing<br>- Surveillance system monitoring<br>- Data quality audits<br>- Surveillance summary reporting<br>- Daily SMS zero-reporting<br>- Field-based mentorship<br>- Goal setting and performance-based learning	- Workshops<br>- Site visits	- Pre- and post-training assessment<br>- Predictive Evaluation framework<br>- Monitoring of SMS reporting rates<br>- post-training interviews	Not certificated	- Limited post-training evaluation due to resources<br>- Language barriers and mentor recruitment<br>- Travel and security issues in remote areas<br>- SMS reporting not implemented in all countries<br>- Self-reported data and non-randomized evaluation
7	Eteng et al	PHEM Fellowship	PHEM Advanced	AfCDC	6 Months	**Thematic areas**<br>-Emergency & Disaster Management<br>-Public Health Science<br>-Leadership & Management<br>**Sub-themes**<br>-Emergency preparedness<br>-PHEOC operations<br>-Epidemiology & surveillance<br>-GIS application<br>-Legal & ethical considerations<br>-Leadership & management<br>-Research<br>-Community engagement<br>-Risk communication	-In person<br>-Online<br>-Hybrid format	- Formative and summative assessments<br>- Practical exams<br>- Peer and mentor evaluations<br>- Self-assessment	Certificated	- Limited existing training opportunities in Africa<br>- Lack of internationally validated PHEM competencies<br>- Language diversity<br>- Resource constraints<br>- Need for contextual adaptation
8	Ario et al	PHFP Fellowship/ FETP	FETP Advanced	MOH, Mak, CDC	24 months	- Response to public health emergencies<br>- Applied epidemiologic studies<br>- Public health surveillance<br>- Communication<br>- Public health programming<br>- Management and leadership	- Classroom based.<br>-Field-based	- Continuous monitoring<br>- Quarterly and annual evaluations<br>- Final portfolio and defense session	Certificated	- Limited recognition of the program<br>- Need for formal accreditation and integration into national systems<br>- Resource constraints for implementing recommended interventions

S/N	Study	Programme Name	Sub-category	Lead Agency	Duration	Competencies Covered	Delivery Method	Evaluation Approach	Certification	Challenges
9	Olu et al	Public Health Disaster Risk Management (DRM) Training Programme	Basic<br>Intermediate<br>Advanced	WHO AFRO, APHTIs	- Basic: 6 days<br>- Intermediate: 14 days<br>- Advanced: 8 weeks field placement	**14 core competencies grouped into 6 thematic areas:**<br>-Introduction to DRM<br>1. Public Health Principles and Practices for DRM<br>2. Basic Epidemiological Methods and Data Management<br>3. Effective Communication in DRM<br>4. Legal Considerations, Human Rights, and Ethics in DRM<br>-Operational Effectiveness<br>5. Resource Mobilization and Management<br>6. Logistics Management<br>7. Safety and Security Measures<br>-Effective Leadership<br>8. Leadership, Teamwork, and Management Skills<br>9. Monitoring and Evaluation Cycle<br>-Preparedness & Risk Reduction<br>10. Capacity Assessments<br>11. Preventive and Mitigation Activities<br>12. Emergency Preparedness at Community and Health Facility Levels<br>-Emergency Response<br>13. Health Response to Disasters and Public Health Emergencies<br>-Post-emergency Health System Recovery<br>14. Health System and Population Recovery	- N/A	- Pre- and post-tests<br>- Written exams<br>- Field reports and oral defense<br>- Participant feedback	Not Certificate	- Limited practical training<br>- Shortage of experienced trainers<br>- Funding constraints<br>- Resistance from institutions<br>- Lack of accreditation modalities across countries
10	Prasarnphanich et al	One Health workforce development tool for zoonotic	-	WHO, FAO, WOAH	Flexible, self-paced	**Technical competencies**<br>-Strategic planning and emergency preparedness<br>-Surveillance and information sharing.<br>-Coordinated investigation and response.	- In-person and online formats	-	Not certificated	- Limited inclusion of environmental sector in pilots<br>- Tool focuses on qualitative assessment,

S/N	Study	Programme Name	Sub-category	Lead Agency	Duration	Competencies Covered	Delivery Method	Evaluation Approach	Certification	Challenges
		disease management				-Joint risk assessment.<br>-Risk reduction,<br>-Risk communication, and community engagement<br>-Research<br>-workforce development.<br>-Monitoring and evaluation<br>**Non-technical competencies**<br>-Leadership<br>-Collaboration<br>-Management				not workforce quantification or inclusivity<br>- Published only in English due to funding constraints
11	Olness et al	Disaster management training with a focus on paediatric care	-	RCIC, CWRU, UNICEF, WHO, AAP, IPA	5 days	-Identifying vulnerable groups in disasters.<br>- Managing infectious diseases<br>- Triage<br>- Psychosocial support<br>- Emergency obstetrics<br>- Understanding humanitarian law<br>- Planning health interventions for refugee groups.<br>-Conducting emergency nutritional assessments	- Didactic classroom based.<br>- Problem-based learn<br>- Case simulations	- Pre- and post-tests<br>- Daily feedback<br>- Final feedback on questionnaire	Not certificated	-Limited faculty expertise in pediatric disaster management<br>-Need for local adaptation of training content<br>-Logistical constraints in resource-limited settings<br>-Time limitations for participants<br>-Sustainability concerns for ongoing training
12	Ntiyaduhanye et al	Integration of One Health topics and competencies into medical education curricula	-	COH, UGHE	3 months	- Epidemiology<br>- Ethics and values<br>- Zoonotic/vector-borne diseases<br>- Risk analysis and biohazard identification<br>- Microbiology (including AMR)<br>- Ecosystem health<br>- Food systems<br>- Comparative medicine<br>- Communication and informatics<br>- Leadership<br>- Policy and advocacy	- Classroom based<br>-Field based<br>-Seminars	-Participants feedback	Not certificated	-Lack of trained faculty in One Health (OH)<br>-Overloaded medical curricula with limited space for OH<br>-Insufficient time for integration<br>-Low institutional prioritization and administrative support

S/N	Study	Programme Name	Sub-category	Lead Agency	Duration	Competencies Covered	Delivery Method	Evaluation Approach	Certification	Challenges
						- Research and systems thinking<br>- Gender, culture, beliefs				-Conceptual unfamiliarity with OH among educators<br>-Concerns about OH distracting from core medical focus<br>-Low emphasis on social science competencies (e.g., gender, culture)
13	Alunyo et al	IDEA Fellowship	-	BU, AfCDC, MOH, UNHRO, IDI, UNIPH, UNHLS, MCRI	24 months	- Epidemiology<br>- Biostatistics<br>- Outbreak investigation<br>- Surveillance<br>- Disease modelling<br>- Bioinformatics<br>- Scientific writing<br>- Public health interventions<br>- Grantsmanship	-Classroom based<br>-Fieldwork, case studies<br>-Research projects	- Survey<br>- Curriculum validation workshops	Certificated	- Limited diagnostic capacity<br>- Weak institutional collaboration<br>- Resource constraints<br>- Inconsistent mentorship<br>- Infrastructure gaps<br>- Policy and governance issues
14	Amuguni et al	Gender, One Health and Emerging Pandemics Threat Short Course	-	Mak, TU, OHCEA, TRG, USAID	5 days	- Gender analysis in disease surveillance and response<br>- One Health principles<br>- Epidemiology of emerging infectious diseases<br>- Emergency response planning<br>- Advocacy and communication	- Practical<br>- Case studies<br>-Simulations<br>-Role plays	-Entry and exit surveys<br>- Participant feedback	Not certificated	- Gender often overlooked in epidemic response<br>- Lack of gender-disaggregated data<br>- Need for systemic integration of gender in public health education and planning
15	Bemah et al	HCWs in ETU, SQS, and RRT Training	-	MOH, NPHIL, WHO, CDC	8 days	- Infection Prevention & Control (IPC)<br>- Ebola case management<br>- Psychosocial support<br>- Surveillance<br>- Emergency response<br>- Safe burials<br>- Rapid response coordination	- Classroom based<br>- Simulations	- Pre- and post-tests<br>- Simulation exercises	Certificated	- Limited data on RRT training impact

S/N	Study	Programme Name	Sub-category	Lead Agency	Duration	Competencies Covered	Delivery Method	Evaluation Approach	Certification	Challenges
16	Kumar et al	FETP	- Advanced<br>- Frontline	MOH, ZNPHI, UNZASPH, CDC	-Advanced-24 months<br>-Frontline-2 months	- Surveillance<br>- Outbreak investigation<br>- Data analysis<br>- Scientific communication<br>- Leadership & management<br>- HIV & malaria surveillance<br>- Emergency response	- Classroom based<br>-Field work	- Program deliverables<br>- Outbreak response participation<br>- Publications and presentations<br>- Strategic planning and stakeholder feedback	- Certificated (Advanced)<br>-Not certificated (frontline)	- Reliance on donor funding<br>- Limited human resources and mentorship capacity<br>- Uneven geographic distribution of graduates<br>- Need for institutionalization and sustainability
17	Subramanian et al	FELTPs	FELTP-Global	TEPHINET, CDC, WHO, AFENET, EMPHNET, SEAFETN, MOH, PHIs	24 months	- Outbreak investigation<br>- Surveillance system development and evaluation<br>- Biostatistics<br>- Analytical and descriptive epidemiology<br>- Communications<br>- Laboratory and veterinary epidemiology	- On-the job learning<br>- Field work<br>- Mentorship	-Membership surveys<br>- Abstract submissions to TEPHINET conferences<br>- Accreditation process	Certificated	- Variation in curricula and host institutions<br>- Need for sustainable funding and institutional support<br>- Accreditation and quality assurance still evolving
18	Kebebew et al	FETP	FETP-Frontline	EPHI, CDC	3 months	- Basic epidemiology<br>- Descriptive statistics<br>- Data management and analysis (Excel)<br>- Surveillance data audit<br>- Case and outbreak investigation<br>- Scientific communication	- On-site training<br>- Field-work<br>- Practical exercise		Not certificated	- Budget constraints<br>- Transportation and logistics shortages<br>- Inadequate mentorship<br>- High staff turnover<br>- Lack of refresher training and career development<br>- Limited federal follow-up
19	Reddy et al	FETP	FETP-Advanced	NICD, NDOH, UP, CDC	24 months	- Epidemiology<br>- Biostatistics<br>- Surveillance system evaluation<br>- Outbreak investigation<br>- Scientific communication	- Mentorship<br>- Field work	- External review<br>- Ongoing impact evaluation<br>- Graduation rate	Certificated	- Low enrolment of medical/veterinary professionals<br>- Delays in MPH thesis completion

S/N	Study	Programme Name	Sub-category	Lead Agency	Duration	Competencies Covered	Delivery Method	Evaluation Approach	Certification	Challenges
						- Public health leadership<br>- Teaching others				- Variable outbreak investigation experiences<br>- Need for standardized career pathways
20	Baltazar et al	FELTP	FELTP-Advanced	INS, MOH, UEM, CDC	-24 months (advanced)<br>-3 months (short course	- Epidemiological methods<br>- Biostatistics<br>- Surveillance system evaluation<br>- Outbreak investigation<br>- Laboratory SOPs<br>- Scientific communication<br>- Leadership and emergency response	-Classroom based<br>- Field placements	- External assessment<br>- Scorecard tool (CDC)<br>- Graduate retention tracking<br>- Output-based performance review	Certificated	- Sustainability and funding<br>- Limited career pathways for graduates<br>- Language barriers in publishing<br>- Need for stronger field placements and mentorship
21	Pokuah et al	FETP	FETP-Frontline	MOH, NPHIL, CDC, AFENET, Emory	3 months	- Integrated Disease Surveillance and Response<br>- Outbreak investigation<br>- Data quality audits<br>- Surveillance reporting and analysis<br>- Scientific communication	- Classroom based<br>- Field projects	- Pre- and post-test scores<br>- post-graduation assessments<br>- IDSR indicator tracking<br>- Stakeholder interviews	Not Certificated	- Limited computer literacy among some trainees<br>- Difficult field access during rainy season<br>- No defined sustainability plan post-CDC funding<br>- Need for career pathways and refresher training
22	SATI	Health Program & Crisis Response Project Management	-	SATI		-Health program design & governance<br>-Crisis response frameworks & leadership<br>-Project planning & implementation<br>-Emergency preparedness & rapid response<br>-Crisis communication & public trust<br>-Health supply chain & logistics<br>-Monitoring & evaluation in emergencies<br>-Leadership under pressure<br>-Risk management & community-based reduction<br>-Stakeholder engagement & donor coordination	-Classroom based	-Not stated	Certificated	-Prepayment<br>-Travel and accommodation costs

S/N	Study	Programme Name	Sub-category	Lead Agency	Duration	Competencies Covered	Delivery Method	Evaluation Approach	Certification	Challenges
						-Humanitarian principles & ethics<br>-Digital innovations (telemedicine, mobile apps, AI)<br>-Financial management & fraud prevention<br>-Health systems resilience<br>-Global case studies (Ebola, pandemics, conflict zones)<br>-Future trends (One Health, climate change, global health security)				
23	André et al	African Public Health Emergency Management Fellowship	-	AfCDC, WHO, CDC, UKHSA, ECDC, BMGF, RKI, RTSL	11 weeks	- Emergency and disaster management<br>- Public health science (epidemiology, surveillance, GIS)<br>- Leadership and management<br>- PHEOC operations<br>- Ethics and legal dimensions	- Classroom based<br>- Group work and case studies	- Mini-seminars for feedback<br>- post-training project implementation	Not certificated	- Limited PHEOC functionality in some countries<br>- Surveillance system gaps<br>- Need for legal frameworks and multi-sectoral coordination
24	Wilson et al	FETP	FETP-Frontline	CDC, MOHs, WHO, AFENET, TEPHINET, DTRA	3 months	- Basic epidemiology<br>- Disease surveillance<br>- Data quality auditing<br>- Outbreak investigation<br>- Case investigation<br>- Surveillance system analysis<br>- Communication and reporting	- Classroom workshops<br>- Fieldwork	- Internal evaluations<br>- Participant feedback<br>- Fieldwork outputs	Certificated	- Difficulty identifying qualified mentors<br>- Risk of trained staff leaving positions<br>- Sustainability dependent on MOH ownership<br>- Costly programme
25	Wilson et al	FETP	FETP-Intermediate	MOH, UW I-TECH, CDC	6 months	- Surveillance system evaluation<br>- Outbreak investigation<br>- HIV program evaluation<br>- Data analysis (Epi Info)<br>- Health communication<br>- Program evaluation	- Classroom based<br>- Field work	- Pre/post-tests<br>- Self-rated competency surveys<br>- Exit interviews<br>- IDSR data audits	Not certificated	- Limited mentor availability<br>- Insufficient time for data analysis practice<br>- Balancing work and field assignments<br>- Need for supervisor engagement
26	Harris et al	FETP	FETP-Advanced	MOH, UNIPH, CDC, MUSPH	24 months	- Outbreak investigation<br>- Surveillance system evaluation<br>- Data analysis<br>- Applied epidemiologic studies	- Field-based full time	- Program database tracking activities<br>- Peer-reviewed publications	Certificated	- Limited regional/district-level engagement early in pandemic

S/N	Study	Programme Name	Sub-category	Lead Agency	Duration	Competencies Covered	Delivery Method	Evaluation Approach	Certification	Challenges
						- Quality improvement<br>- Leadership<br>- Cost analysis<br>- Emergency response		- Real-time response outputs<br>- Expert opinion and stakeholder feedback		- Fellows overloaded with multiple projects<br>- No dedicated post-program job track
27	Nguku et al	FELTP	FELTP-Advanced	FMOH, FMARD, CDC, AFENET, UI, ABU	24 months	- Epidemiology<br>- Public health surveillance<br>- Biostatistics<br>- Scientific communication<br>- Veterinary epidemiology<br>- Laboratory management<br>- Leadership and teaching	- In-service training<br>- Field placements	-Dissertation defence<br>- Cluster exams<br>- Field project outputs<br>- Surveillance system evaluations<br>- Operational research	Certificated	- Sustainable funding<br>- Career progression and retention<br>- Weak surveillance systems<br>- Need for more epidemiologists
28	Roka et al	FETP	FETP-Frontline	MOH, CDC, KEMRI, MoALD, CHMT	3 months	- Data quality assessment (DQA)<br>- Data consistency assessment (DCA)<br>- On-time reporting (OTR)<br>- Surveillance<br>- Epidemiology<br>- Data analysis<br>- Field investigations<br>- Communication	-Classroom based<br>-Field work	- Repeated measures post-training<br>- Online surveys	Not certificated	- Limited impact on data consistency across levels<br>- Self-reported data limitations<br>- Time gap between training and evaluation<br>- No diploma may affect uptake among younger professionals
29	ATI	Public Health in Humanitarian Crises	-	ATI	Flexible self-paced	-Humanitarian disasters and public health<br>-Humanitarian actors and coordination<br>-Water, sanitation, and hygiene (WASH)<br>-Livelihoods and food security<br>-Nutrition in crises<br>-Disease management in emergencies<br>-Health care delivery in humanitarian settings<br>-Shelter provision<br>-Protection of displaced populations, human rights, and humanitarian law	-Classroom based (online)	-Not mentioned	Not certificated	-Limited Slots<br>-Payment confirmation as prerequisite

S/N	Study	Programme Name	Sub-category	Lead Agency	Duration	Competencies Covered	Delivery Method	Evaluation Approach	Certification	Challenges
						-Emerging risks and future challenges				
30	Oyugi et al	Regional Training Centre for the Emergency Medical Teams Initiative	-	WHO, MOH	12 months	- EMT awareness and induction<br>- Case management<br>- WASH and logistics<br>- SOP development<br>- Pre-deployment readiness<br>- Training of Trainers (ToT)<br>- Surveillance	- Classroom based<br>- Field work	- M&E framework<br>- Operational readiness<br>- Feedback and performance tracking	Not certificated	-Staffing shortages for M&E<br>- Infrastructure and procurement delays<br>- Inter-agency coordination issues<br>- Limited simulation resources in some countries
31	Conteh et al	Strengthening and Utilizing Response Groups for Emergencies Flagship Initiative	-	WHO AFRO and AfCDC	24 months	- Public Health Emergency Operations<br>- Incident Management System<br>- Humanitarian Coordination<br>- Rapid Response Teams<br>- IPC, Laboratory, RCCE, GBV, PSEAH<br>- Case Management<br>- Surveillance	- Online training<br>- Face-to-face onboarding training	- Pre- and post-tests<br>- Daily evaluations and feedback<br>- Facilitator debriefs<br>- Bi-weekly progress meetings<br>- KPI tracking via dashboard	Not certificated	- Inadequate financial resources<br>- Delays in roadmap approval and trainee identification<br>- Limited budget to train anticipated numbers<br>- Need for sustained funding and donor engagement
32	Ameme et al	FELTP	-	UG; GHS; VSD	12 weeks	- Public health surveillance<br>- Outbreak investigation and response<br>- Surveillance system evaluation<br>- Data analysis for public health action<br>- Use of Excel and Epi Info software	- Didactic classroom sessions<br>- Case studies<br>- Fieldwork<br>- Presentations	- Pre and post-tests<br>- Participant feedback<br>- Quality of assignments (written/oral)<br>- Fieldwork performance and dissemination workshop	Not certificated	- Participants want longer training period<br>- Limited prior capacity among frontline workers to respond to public health emergencies

Acronyms

**CWRU**	Case Western Reserve University (US)
**AAU-SPH**	Addis Ababa University School of Public Health (Ethiopia)
**ABU**	Ahmadu Bello University (Nigeria)
**AfCDC**	Africa Centres for Disease Control and Prevention
**AFENET**	African Field Epidemiology Network
**APHIs**	African public health training institutions
**ATI**	Africa Training Institute
**B&MGF**	Bill and Melinda Gates Foundation
**BU**	Busitema University (Uganda)
**CHMT**	County Health Management Team (Kenya)
**COH**	Centre for One Health
**AAP**	American Academy of Pediatrics
**DTRA**	Defense Threat Reduction Agency
**ECDC**	European Centres for Disease Prevention and Control
**EHNRI**	Ethiopian Health and Nutrition Research Institute
**Emory**	Emory University (US)
**EMPHNET**	Eastern Mediterranean Public Health Network
**EPHA**	Ethiopian Public Health Association
**EPHI**	Ethiopian Public Health Institute
**FELTP**	Field Epidemiology and Laboratory Training Programme
**FETP**	Field Epidemiology Training Programme
**FMARD**	Federal Ministry of Agriculture and Rural Development (Nigeria)
**FMOH**	Federal Ministry of Health (Ethiopia)
**GHS**	Ghana Health Services
**HCWs in ETU, SQS, and RRT Training**	Healthcare Workers in Ebola Treatment Unit, Safe & Quality Services, and Rapid Response Team Training
**ICAP**	International Center for AIDS Care and Treatment Programs (United States)
**IDEA Fellowship**	Infectious Disease Epidemiology and Biostatistics in Africa Fellowship (Uganda)
**IDI**	Infectious Diseases Institute (Uganda)
**INS**	National Institute of Health (Mozambique)
**IPC**	Infection Prevention and Control
**KEMRI**	Kenyan Medical Research Institute
**MCRI**	Mbale Clinical Research Institute (Uganda)
**MoALD**	Ministry of Agriculture, Livestock and Fisheries (Kenya)
**MUSPH**	Makerere University School of Public Health (Uganda)
**NDOH**	National Department of Health (South Africa)
**NICD**	National Institute for Communicable Diseases (South Africa)
**NPHIL**	National Public Health Institute of Liberia
**OHCEA/AFROHUN**	One Health Central and Eastern Africa/ Africa One Health University Network

**PEPFAR**	President's Emergency Plan for AIDS Relief
**PHEM**	Public Health Emergency Management
**PHEOC**	Public Health Emergency Operations Center
**PHFP Fellowship**	Public Health Fellowship Program (Uganda)
**PHIs**	Public Health Institutes
**PMI**	President's Malaria Initiative
**RCIC**	Rainbow Center for International Child Health
**RKI**	Robert Koch Institute (Germany)
**RRT**	Rapid Response Team
**RTSL**	Resolve to Save Lives
**SATI**	Skills for Africa Training Institute
**SEAFETN**	Southeast Asia Field Epidemiology Training Network
**STEP**	Surveillance Training for Ebola Preparedness
**TEPHINET**	Training Programs in Epidemiology and Public Health Interventions Network
**TRG**	Training Resource Group
**TU**	Tufts University (US)
**UEM**	Eduardo Mondlane University (Mozambique)
**UG**	University of Ghana
**UGHE**	University of Global Health Equity (Rwanda)
**UI**	University of Ibadan (Nigeria)
**UKHSA**	UK Health Security Agency
**UNHLS**	Uganda National Health Laboratory Services
**UNHRO**	Uganda National Health Research Organisation
**UNIPH**	Uganda National Institute of Public Health
**UP**	University of Pretoria (South Africa)
**USAID**	United States Agency for International Development
**UW I-TECH**	University of Washington International Training and Education Centre for Health (USS
**UZASPH**	University of Zambia School of Public Health
**VSD**	Veterinary Services Division (Ghana)
**WOAH**	World Organization for Animal Health
**ZNPHI**	Zambia National Public Health Institute
