# Supplementary material for: Scoping review of public health emergency management training programmes and curricula in Africa (2000–2025): implications for policy, practice and research
Source: BMJ Public Health. 2026 Sep 21;4(Suppl 1):e004980. doi: 10.1136/bmjph-2026-004980 (PMC13599839; doi:10.1136/bmjph-2026-004980)
Supplement: Supplementary file 3 [file bmjph-4-Suppl_1-s003.pdf]

Supplementary material 3: Summarized characteristics of included studies

N	Author	Year	Study Title	Design	Location	Source	Target Audience	Key Focus
1	Jima et al [24]	2011	The Ethiopian Field Epidemiology and Laboratory Training Program: strengthening public health systems and building human resource capacity	Mixed Methods	Ethiopia	JA	FELTP Residents	FETP – system strengthening
2	Gatei et al [25]	2018	Field Epidemiology and Laboratory Training Program, Where Is the L-Track?	Mixed methods	Kenya	JA	FELTP residents from national and regional labs	FETP – lab track gap
3	Kebebew et al [26]	2022	A comparative cross-sectional evaluation of the Field Epidemiology Training Program-Frontline in Ethiopia	Cross-sectional	Ethiopia	JA	District-level surveillance officers	FETP-Frontline evaluation
4	Mbola et al [27]	2024	Disaster management training for environmental health: A narrative literature review	Narrative lit review	ESA	JA	Environmental health practitioners (Disaster responders)	Disaster training environmental health
5	Siouris et al [28]	2022	Rapid scale-up of COVID-19 training for frontline health workers in 11 African countries	Mixed methods	Africa	JA	Frontline HCWs (facility-based COVID-19 responders)	COVID-19 Training Scale-up

6	Cáceres et al [29]	2017	Surveillance Training for Ebola Preparedness in Côte d'Ivoire, Guinea-Bissau, Senegal, and Mali	Mixed methods	West Africa	JA	District surveillance officers in Ebola-border regions	EVD surveillance training
7	Eteng et al [15]	2024	Designing a competency-based curriculum for an advanced training program in public health emergency management: a stepwise, mixed method approach, 2023-	Mixed methods	Africa	JA	Africa CDC PHEM fellows	PHEM curriculum design
8	Ario et al [30]	2018	Strengthening Global Health Security Through Africa's First Absolute Post-Master's Fellowship Program in Field Epidemiology in Uganda	Descripti ve	Uganda	JA	PHFP fellows	FETP fellowship – GHS
9	Olu et al [12]	2018	What should the African health workforce know about disasters? Proposed competencies for strengthening public health disaster risk management education in Africa	Mixed methods	Africa	JA	African health workforce involved in disaster risk management	DRM competenci es
10	Prasarnp hanich et al [31]	2025	Developing a Tripartite (Food and Agriculture Organization of the United Nations; World Health Organization; and World Organisation for Animal Health) tool to strengthen the workforce for effective management of zoonotic diseases	Mixed methods	Africa	JA	One Health workforce	Tripartite tool – zoonoses workforce
11	Olness [32]	2005	Training of Health Care Professionals on the Special Needs of Children in the Management of Disasters: Experience in Asia, Africa, and Latin America	Mixed methods	Africa+	JA	HCWs (trained in pediatric disaster response)	Child-focused disaster training

12	Ntiyaduhanye et al [33]	2024	One Health in sub-Saharan African medical curricula: a cross-sectional, mixed-methods study	Mixed methods	SSA	JA	Medical students	One Health – curricula integration
13	Alunyo et al [34]	2025	Capacity Building in Field Epidemiology in Sub Saharan Africa: Findings from Infectious Disease Field Epidemiology and Biostatistics in Africa (IDEA) Fellowship Program	Cross-sectional	SSA	JA	IDEA fellows	IDEA fellowship – FETP capacity
14	Amuguni et al [35]	2018	EnGENDERing One Health and addressing gender gaps in Infectious disease control and response: Developing a Gender, One Health and Emerging Pandemics threat short course for the public health workforce in Africa.	Descriptive	Africa	JA	Gender & One Health trainees	Gender–One Health training
15	Bemah et al [36]	2019	Strengthening healthcare workforce capacity during and post Ebola outbreaks in Liberia: an innovative and effective approach to epidemic preparedness and response	Cross-sectional	Liberia	JA	EVD Trained HCWs in Liberia	EVD response – workforce strengthening
16	Kumar et al [37]	2020	Zambia field epidemiology training program: strengthening health security through workforce development	Descriptive	Zambia	JA	FETP Trainees	FETP – health security
17	Subramanian et al [38]	2013	An evaluation of the global network of field epidemiology and laboratory training programmes: a resource for improving public health capacity and increasing the number of public health professionals worldwide	descriptive	AFRICA+	JA	FETP/EIS trainees	FETP network – capacity building
18	Kebebew et al [39]	2023	Evaluation of Ethiopia's field epidemiology training program – frontline: perspectives of implementing partners	Cross-sectional	Ethiopia	JA	FETP-Frontline trainees (District	FETP-Frontline – Evaluation

							Surveillance Officers)	
19	Reddy et al [40]	2019	South Africa field epidemiology training program: developing and building applied epidemiology capacity, 2007–2016	Descriptive	South Africa	JA	FETP residents (MPH-level applied epidemiologists)	FETP – capacity building
20	Baltazar et al [41]	2017	Mozambique field epidemiology and laboratory training program: a pathway for strengthening human resources in applied epidemiology	Descriptive PR	Mozambique	JA	FELTP trainees	FETP – HR strengthening
21	Pokuah et al [42]	2021	The Frontline Field Epidemiology Training Program in Liberia, 2015-2018	Descriptive	Liberia	JA	FETP-F trainees	FETP-Frontline – rollout
22	Skills for Africa Training Institute [43]	2025	Health Program & Crisis Response Project Management Training Course	N/A	AFRICA	GL	Multidisciplinary health & crisis response professionals	Crisis response – PM
23	Getaneh [16]	2025	The Africa Centres for Disease Control public health emergency management fellowship: insights from the inaugural cohort	Descriptive	AFRICA	JA	Africa CDC PHEM fellows	AfCDC PHEM fellowship – cohort insights
24	André [44]	2017	Frontline Field Epidemiology Training Programs as a Strategy to Improve Disease Surveillance and Response	Descriptive PE	West Africa	JA	FETP-Frontline trainees	FETP-Frontline – surveillance

								strengthening
25	Wilson et al [45]	2021	Evaluation of a New Field Epidemiology Training Program Intermediate Course to Strengthen Public Health Workforce Capacity in Tanzania	Pre/post evaluation	Tanzania	JA	FETP-Intermediate trainees	FETP-Intermediate – Evaluation
26	Harris et al [46]	2022	Improving the effectiveness of Field Epidemiology Training Programs: characteristics that facilitated effective response to the COVID-19 pandemic in Uganda	Descriptive case study	Uganda	JA	FETP-Advanced Fellows	FETP – COVID-19 response enablers
27	Nguku et al [47]	2014	Training and Service in Public Health, Nigeria Field Epidemiology and Laboratory Training, 2008 – 2014	Descriptive PR	Nigeria	JA	FELTP trainees	FETP – training & service integration
28	Roka et al [48]	2021	Impact Evaluation of the Kenya Frontline Field Epidemiology Training Program: Repeated-Measures Study	Mixed methods	Kenya	JA	FETP-Frontline trainees	FETP-Frontline – impact evaluation
29	Africa Training Institute [49]	No date	Public Health in Humanitarian Crisis	N/A	AFRICA	GL	Humanitarian Health Responders	Humanitarian crisis – public health systems
30	Oyugi et al [50]	2025	The regional training centre for the emergency medical teams initiative in the WHO African region: a review of the development and progress over the past 4 years	Descriptive	AFRO	JA	EMT/RRT trainees	WHO AFRO EMTs Training review

31	Conteh et al [13]	2024	Strengthening and utilizing response groups for emergencies flagship: a narrative review of the roll out process and lessons from the first year of implementation	Descriptive	AFRO	JA	AVoHC-SURGE responders	Emergency response groups Flagship rollouts lessons
32	Ameme et al [51]	2016	Training Ghanaian frontline healthcare workers in public health surveillance and disease outbreak investigation and response	Pre-test and post-test intervention study	Ghana	JA	FELTP-frontline trainees	Frontline Training in outbreak surveillance & Response
